# Combating Bacterial Biofilm Formation in Urinary Catheter by Green Silver Nanoparticle

**DOI:** 10.3390/antibiotics11040495

**Published:** 2022-04-08

**Authors:** Reham M. Goda, Ahmed M. El-Baz, Eman M. Khalaf, Nada K. Alharbi, Tarek A. Elkhooly, Mohamed M. Shohayeb

**Affiliations:** 1 Department of Microbiology and Biotechnology, Faculty of Pharmacy, Delta University for Science and Technology, Gamasa 11152, Egypt; dr_reham_magdy@yahoo.com (R.M.G.); shohayeb@hotmail.com (M.M.S.); 2 Department of Microbiology and Immunology, Faculty of Pharmacy, Damanhour University, Damanhour 22511, Egypt; eimanpharmacist@gmail.com; 3 Department of Biology, College of Science, Princess Nourah bint Abdulrahman University, P.O. Box 84428, Riyadh 11671, Saudi Arabia; nkalharbi@pnu.edu.sa; 4 Nanomedicine Research Unit, Faculty of Medicine, Delta University for Science and Technology, Gamasa 11152, Egypt; tarek.elkhooly@gmail.com; 5 Department of Refractories, Ceramics and Building Materials, National Research Centre, Giza 12622, Egypt; 6 Physics Department, Faculty of Science, New Mansoura University, New Mansoura City 35511, Egypt

**Keywords:** silicon urinary catheters, biofilm, mastic, silver nanoparticles, *Pistacia lentiscus*, *Punica granatum*

## Abstract

Urinary catheters are commonly associated with urinary tract infections. This study aims to inhibit bacterial colonisation and biofilm of urinary tract catheters. Silicon catheter pieces were varnished with green silver nanoparticles (AgNPs) using *Pistacia lentiscus* mastic to prevent bacterial colonisation. Pomegranate rind extract was used to synthesize AgNPs. AgNPs were characterized by UV-Vis spectroscopy, X-ray crystallography, and transmission electron microscopy (TEM). Results obtained revealed that the size of most AgNPs ranged between 15–25 nm and they took crystallised metal and oxidised forms. The amounts of released silver ions from 1 cm pieces of catheters coated with AgNPs were estimated for five days and ranged between 10.82 and 4.8 µg. AgNPs coated catheters significantly inhibited the colonisation of catheters by antibiotic-resistant clinical Gram-positive (*Staphylococcus epidermidis* and *Staphylococcus aureus*) and Gram-negative (*Escherichia coli*, *Klebsiella pneumoniae*, *Proteus mirabilis*, and *Pseudomonas aeruginosa*) bacteria. AgNPs-varnish was more active against Gram-negative bacteria than Gram-positive bacteria. The significant inhibitory effect of coated catheters lasted for 72 h for both Gram-positive and Gram-negative bacteria. Varnishing catheters with AgNPs may help to prevent bacterial colonisation and infections.

## 1. Introduction

Urinary tract infections (UTIs) are the most common infections in community and hospital settings [1,2]. In hospitals, UTIs account for 40–50% of nosocomial infections [3]. This leads to longer hospital stays and an increase in medical costs [4]. Around 15–25% of hospitalized patients receiving indwelling urinary catheters develop catheter-associated urinary tract infections (CAUTIs) [5].

Urinary catheters are tubular latex or silicone devices. Inserted catheters may readily acquire biofilms on their inner or outer surfaces. The capability of microorganisms to form catheter biofilms is directly proportional to the time for which the catheter remains without change [6]. Biofilm formation is a process in which microorganisms irreversibly attach to and grow on a surface and produce extracellular polymers that facilitate attachment and matrix formation [7].

The most common biofilm-forming bacteria causing CAUTIs are *E. coli*, *Kl. pneumoniae*, *Enterococcus faecalis*, *Pr. mirabilis*, *Staph. aureus*, *Staph. epidermidis*, *Ps. aeruginosa* and *Candida* spp. [8,9]. *E. coli*, *Staphylococcus* spp., and *Klebsiella* spp. account for 75–90%, 5–15%, and 4.3–7% of uncomplicated cases of CAUTIs [10].

The adhesion of microorganisms to catheter materials is mainly dependent on the hydrophobicity of both the organisms and the catheter surface [11]. Moreover, the attachment and colonisation of bacteria to the surface of catheters is enhanced by its increased ionic strength due to the presence of divalent cations such as calcium and magnesium in urine and its relatively acidic pH [12]. Bacteria forming biofilms are usually resistant to antibiotics. In addition, biofilms hinder the penetration of antibiotics and create an unfavourable chemical microenvironment for antibiotics to be fully active [13]. Various methods have been developed to prevent bacteria colonisation of catheters. These include the addition of antimicrobial agents in collection bags, the use of antimicrobial ointments and lubricants, and the use of systemic antibiotics [14]. However, most of these strategies have not been fully effective [15].

Silver has been widely used for the treatment of microbial infections [16], in the form of wound dressings [17], medical textiles [18], and as coatings for medical devices [19,20].The ability of silver ions (Ag^+^) to kill bacteria has long been established [21,22], where, Ag^+^ kills bacteria by damaging their cell membranes [23], interacting with macromolecules such as proteins and DNA, and inactivating essential enzymes [21,24]. Silver in the form of an alloy along with gold or platinum or nanoparticles is coated onto both the external and internal surfaces of the catheters. Bardex^®^ IC and DoverTM, both approved by the Food and Drug Administration (FDA), are the two most commonly used and studied silver Foley catheters [25]. Silver-coated catheters showed significant in vitro prevention of biofilms of *E. coli*, *Enterococcus*, *Staph. aureus*, coagulase-negative staphylococci, *Ps. aeruginosa*, and *C. albicans* [26]. Studies suggest that silver-coated catheters successfully reduce infection rates and catheter-associated bacteriuria [27]. a mixture of oxidized and metallic AgNPs exhibit higher antibacterial activity in bacterial cultures than only metallic silver forms because of the higher dissolution rate [28].

In this study, green silver nanoparticles were prepared by pomegranate (*Punica granatum*) rind extract and were varnished on the surface of pieces of catheters using the resin of *Pistacia lentiscus*. The resin of *P*. *lentiscus* possesses antiviral, antibacterial, and antifungal capabilities [29]. In a previous study, *P. lentiscus* resin was used to varnish catheters with benzoic acid and chlorhexidine. The varnish in vitro slowly released the two antimicrobials and inhibited the colonisation of catheters by both Gram-positive and Gram-negative bacteria [30].

## 2. Results

**Figure 1:**
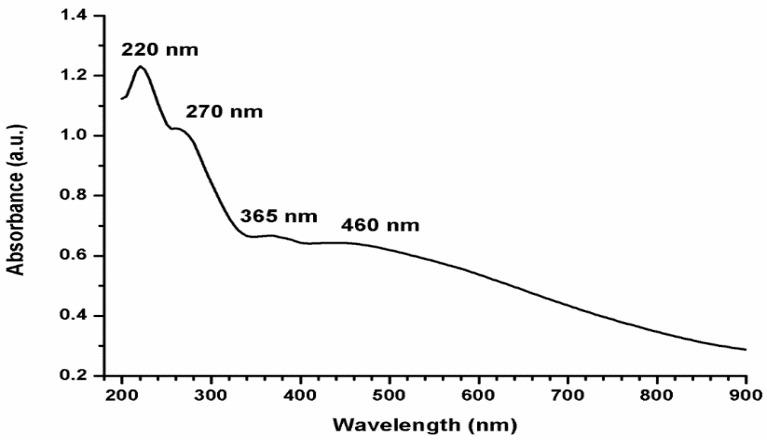
UV–Vis spectrum of AgNPs prepared by pomegranate peel extract in the wavelength range of 200–900 nm.

The reduction of the Ag^+^ to AgNPs using pomegranate rind extract was associated with a colour change from pale pink to dark brown colour. The UV–visible spectrum of AgNPs was recorded in the wavelength range of 200–900 nm. Figure 1 shows three sharp peaks 220, 270, and 365. These peaks are attributed to the π–π* electronic transitions of the organic compound in the extract. The broader peak detected at 460 nm for AgNPs indicates the anisotropic properties of the size and shape of reduced AgNPs.

**Figure 2:**
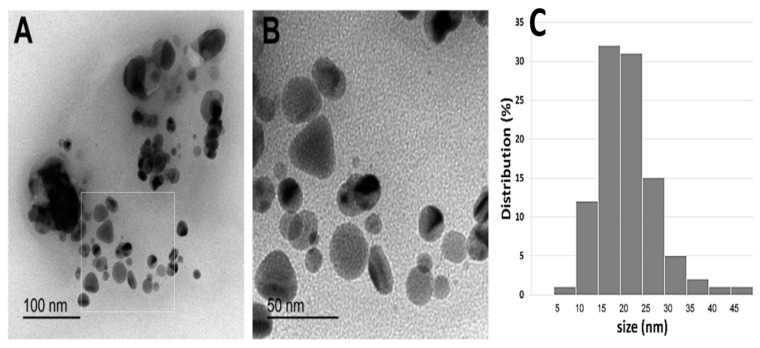
(**A**,**B**), different shapes and sizes of silver nanoparticles as detected by TEM; (**C**) particle size distributions.

Different shapes of silver nanoparticles were detected in TEM micrographs as shown in Figure 2A,B. Spherical and nearly spherical nanoparticles were the most abundant shapes in five different micrographs with sizes ranging from 5–50 nm. Rods and triangles were also detected, but they were less common (Figure 2). AgNPs tended to form agglomerates as observed in Figure 2A.

**Figure 3:**
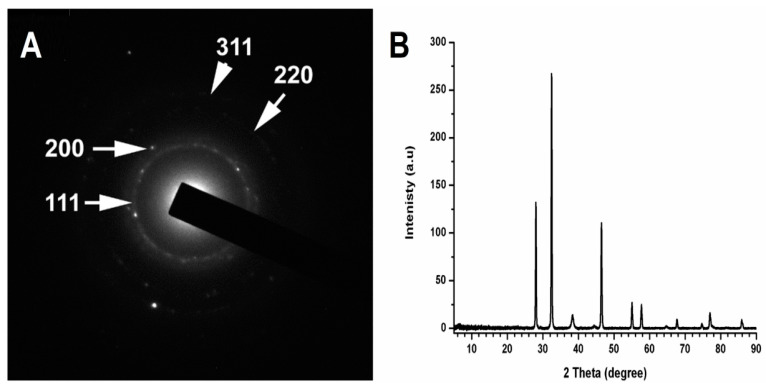
(**A**) Selected area electron diffraction (SAED) pattern analysis of AgNPs; (**B**) XRD pattern of AgNPs.

The composition and crystallinity of the AgNPs were analysed by electron diffraction (SAED) and X-ray Diffraction (XRD). As shown in Figure 3, there are four very narrow diffraction peaks at 111, 200, 220, and 311 (Figure 3A), which suggest crystalline forms of metallic AgNPs.

The crystalline nature of nanoparticles was also confirmed by X-ray crystallography. The diffracted intensities were recorded from 2θ = 10 to 90. The XRD pattern of the synthesized AgNPs is shown in Figure 3B. The XRD diffraction peaks corresponding to 2θ = 38.45, 46.35, 64.75, and 78.05 were assigned to diffractions from (111), (200), (220), and (311) planes, respectively, which correspond to crystalline elemental Ag (JCPDS 87-0717). XRD pattern of AgNPs also indicates AgNPs in the form of silver oxides (2θ values equal to 32.24, 55.04, and 57.870). The oxides of AgNPs at 2θ values equal to 32.24 were more predominant than other peaks (Figure 3B).

**Figure 4:**
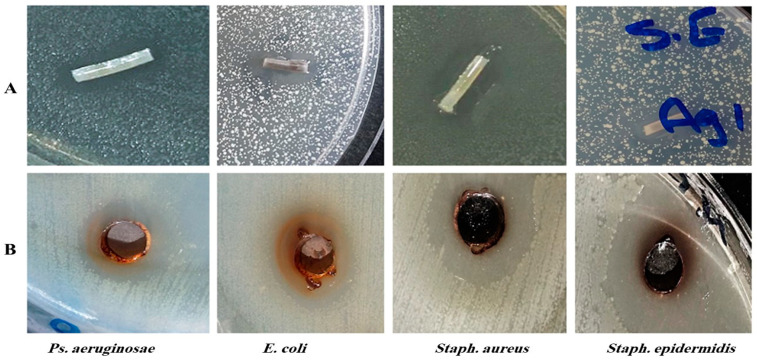
The inhibitory effect of catheters impregnated in AgNPs/mastic varnish on the different tested bacteria. (**A**) Top figures represent the inhibition zones produced by the pieces of the varnished catheters. (**B**) The bottom figures represent the inhibition of bacteria by AgNPs by the cup–plate method.

AgNPs were tested for their antimicrobial activity against six antibiotic-resistant and biofilm-forming clinical bacterial isolates by the cup–plate method. All isolates were inhibited by AgNPs. Figure 4A demonstrates the inhibition zones produced by the pieces of the varnished catheters against streaked bacteria. Figure 4B represent the inhibition of the streaked bacteria by AgNPs by the cup–plate method.

Pieces of urinary catheter coated with silver nanoparticles were tested for their antimicrobial activity against the six tested biofilm-forming bacteria. Figure 4B shows a zone of inhibition of 15 mm around a piece of catheter applied onto a plate seeded with *Staph. epidermidis*. 

**Figure 5:**
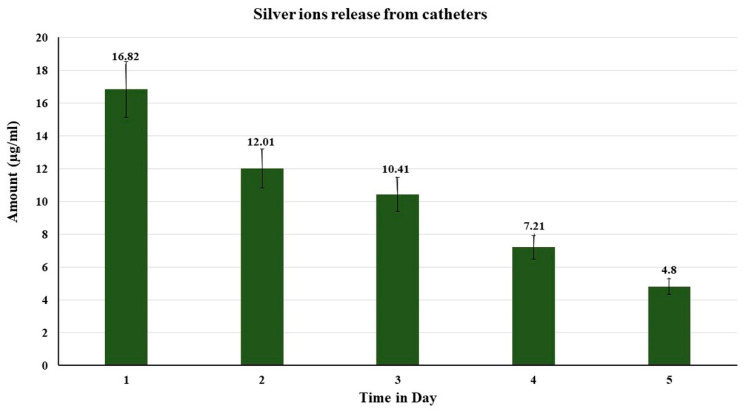
The amounts of silver ion released from catheters coated with silver nanoparticles and varnished with mastic from day 1 to 5.

The amount of silver ions released was sustained for a duration of five days as shown in Figure 5. The amount of silver ions released ranged between 16.82 µg on day one and 4.80 µg on day five.

**Figure 6:**
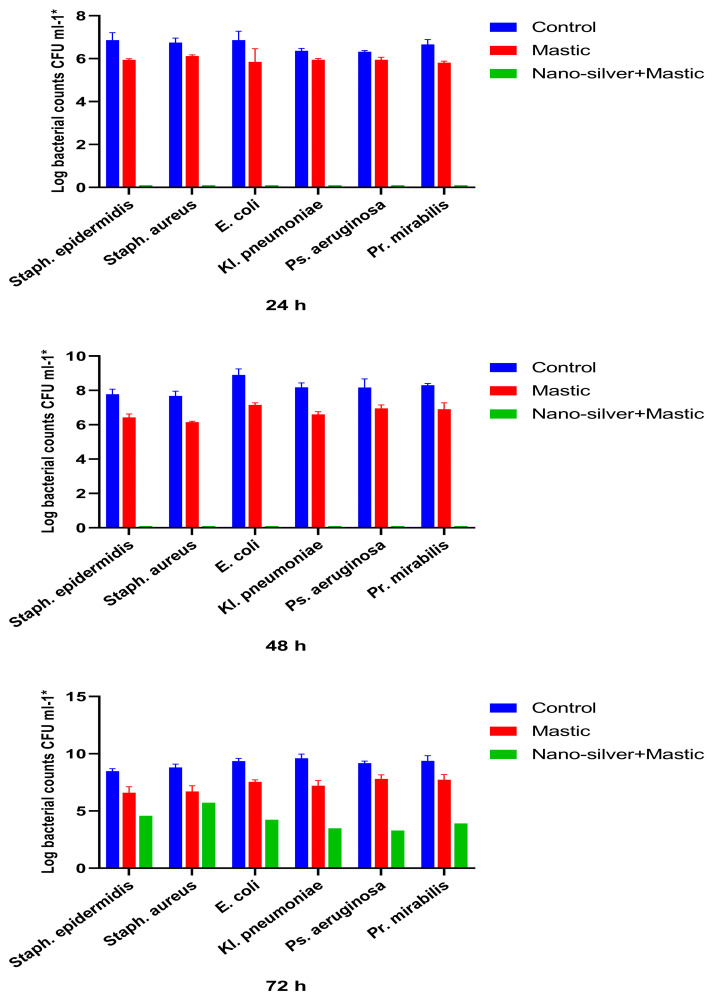
Inhibitory effects of silver nanoparticles and *Pistacia lentiscus* on the adherence of different types of bacterial cells to the surface of 1 cm silicon catheter.

The inhibitory effect of the colonisation of urinary catheters varnished with AgNPs on biofilm-forming bacteria was evaluated by the viable count technique after the incubation of catheters with each of the tested bacteria in nutrient broth, for 24 h, 48 h, and 72 h. Figure 6 summarises the viable counts of the tested bacteria colonising the untreated catheter, catheter varnished with mastic only, and catheter varnished with both mastic and silver nanoparticles.

It was noticed that the varnish of the mastic alone without silver nanoparticles exhibited a moderate inhibitory effect on the colonisation of the catheter by the tested colonising bacteria (Figure 6).

The AgNPs-coated catheters completely inhibited colonisation of the catheter pieces by all the six tested bacteria in the first 48 h (Figure 6). After 72 h, all the tested bacteria started to colonise the coated catheters, but the counts of colonising bacteria were significantly (*p ≤* 0.05) less than those of the control. The reduction in counts ranged between 3.09 and 6.13 logs (Figure 6).

The inhibitory effect of AgNPs-varnish was significantly (*p ≤* 0.05) more prominent in Gram-negative bacteria than Gram-positive bacteria (Figure 6). After 72 h, while the decrease in the log count of Gram-negative bacteria ranged between 5.13 and 6.13 logs, the decrease in Gram-positive bacteria ranged between 3.09 and 3.90 logs (Figure 6).

**Figure 7:**
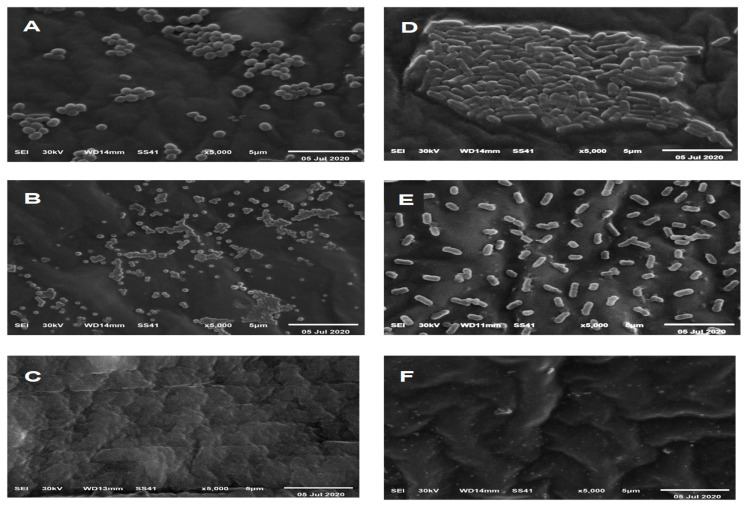
Electron micrographs of the bacterial colonisation of control catheters (**A**,**D**), catheters varnished with mastic (**B**,**E**), and catheters varnished with mastic and AgNPs (**C**,**F**) after 48 h impregnation in nutrient inoculated with 10^5^ CFU mL^−1^ of *Staph. aureus* (**A**–**C**) and *Kl. pneumoniae* (**D**–**F**).

Bacterial colonisation of control catheters, catheters varnished with mastic alone, and catheters varnished with mastic-AgNPs were examined by scanning electron microscopy (Figure 7). After 48 h, bacteria colonising the control catheters appeared in clusters of *Staph. aureus* (Figure 7A) and stacked rods in the case of *Kl. pneumoniae* (Figure 7D). Lower numbers of colonising bacterial cells were detected on catheters coated with mastic alone. Some cells were smaller in size and produced protrusion and polypi (Figure 7B,E). AgNPs-mastic coats completely inhibited the colonisation of the catheter by *Kl. pneumoniae* and *Staph. aureus* (Figure 7C,F).

## 3. Discussion

CAUTIs may lead to serious complications and significant morbidity and mortality [31]. To prevent the colonisation of urinary catheters by pathogens, antimicrobial agents were used as coats for catheters or were incorporated into the catheter materials [32]. One of the most commonly used antimicrobial agents in medical devices to prevent microbial colonisation is silver. Ag exhibits antimicrobial activity against both antibiotic-sensitive and resistant Gram-positive and Gram-negative bacteria [16,31,33].

In this study, AgNPs were prepared using the extracts of the rind of the fruit of the pomegranate. This extract possesses phytochemicals, such as ellagitannins, which have moderate antibacterial activity against *E. coli*, *Kl. pneumoniae*, *Bacillus subtilis*, *Ps. aeruginosa*, and *Pr. mirabilis* [34].

The UV–Vis spectrum of AgNPs exhibited broadband at 450 nm probably due to the formation of AgNPs with different shapes [35]. The shape, size, and uniformity of green AgNPs usually depend on the type of plant extract used [35].

The prepared AgNPs were mostly spherical or nearly spherical. Their size ranged from 5 to 30 nm. They tended to form agglomerates probably due to the capping agents of the phytochemicals found in the extract [36].

X-ray crystallography and the XRD pattern of the synthesized AgNPs confirmed the crystalline nature of AgNPs (2θ = 38.45, 46.35, 64.75 and 78.05) and the presence of silver oxides (2θ = to 32.24, 55.04 and 57.870) as previously reported [37,38,39].

The antimicrobial effect of the silver nanoparticles was confirmed by using the SEM technique which showed a significant reduction in bacterial counts. The results of the current study are consistent with another recent study that reported the antimicrobial activity of green AgNPs [40].

The catheter was coated with silver nanoparticles using a varnish of *P. lentiscus* mastic. This mastic is known to have moderated antibacterial activity [29,30]. Therefore, the mastic was used as an adhesive to varnish AgNPs onto the pieces of silicon urinary catheters.

The amounts of silver ion released from the coated catheters were sustained for a duration of at least 5 days and ranged between 16.82 µg on day one and 4.80 µg on day five. The level of silver ions released in this study are bacteriostatic for both Gram-positive and Gram-negative bacteria on the light of previous results [41,42]. A higher concentration of 20 μg/mL silver ion is required to kill bacteria [43].

Silver-coated catheters were challenged with four Gram-negative bacteria (*E. coli*, *Ps. aeruginosa*, *Pr. mirabilis*, *and Kl. pneumoniae*) and two Gram-positive bacteria (*Staph. aureus* and *Staph. epidermidis*). All the tested bacteria are common in CAUTIs [44].

AgNPs have an increased surface area that leads to the release of more Ag^+^ [45]. The antibacterial experiments in this study indicated that catheters varnished with AgNPs produced inhibition zones in Mueller–Hinton agar plates inoculated with the six tested microorganisms.

In this study, mastic alone without silver nanoparticles exhibited moderate inhibition on the colonisation of the catheter. The decrease in the log CFU by mastic was obvious when the catheters were examined by the SEM.

Viable count experiments also revealed a reduction in the bacterial viable counts colonising the pieces of catheters varnished with mastic alone. The decreases in counts ranged between 0.41 and 2.41 logs. The decreases in counts were generally insignificant except in the case of *Kl. pneumonia.* The reduction in the viable counts of *Kl. pneumonia* after 48 and 72 h was ≥2 log (≥99%). Therefore, the mastic probably acted as an adhesive for AgNPs and augmented the antibacterial activity of the silver-nanoparticle-coat.

The varnished AgNPs completely inhibited the colonisation of the catheter by all tested bacterial strains for 48 h. Although bacteria after 72 h started to colonise the pieces of catheters, their counts were still significantly (*p ≤* 0.05) less than the counts of the control by 2.55 to 6.13 log.

It was also observed that the inhibitory effect of the AgNPs layer was more prominent in Gram-negative bacteria compared to Gram-positive bacteria. These data are consistent with the previous study of Paredes and his colleagues [33].

## 4. Material and Methods

### 4.1. Media and Chemicals

Oxoid agar, nutrient broth, Mueller––Hinton agar, and tryptic soy broth were purchased from Oxoid (Hampshire, UK). Other chemicals were of pharmaceutical grade.

### 4.2. Preparation of AgNPs 

Fifteen grams of clean shredded pomegranate peel was added to 200 mL of distilled water in a 500 mL flask. The flask was heated in a water bath at 85 °C for 30 min. The obtained raw extract was cooled and filtered. An amount of 50 mL of pomegranate peel extract was mixed with 100 mL of AgNO_3_ (5 mmol L^−1^) solution at room temperature. The mixture was magnetically stirred for 20 min at room temperature and then placed in a water bath at 35 °C. The colour of the solution turned slowly from light yellow to dark brown. The colloidal solution was centrifuged at high speed for 10 min. The precipitated silver nanoparticles were washed with 95% ethanol solution and water, respectively, and were dried at 150 °C for 8 h [39].

### 4.3. Characterisation of AgNPs

UV spectroscopy: Several diluted solutions of AgNPs were examined by using UV–vis spectrophotometer (Jasco, V-730, Tokyo, Japan) at a wavelength range of 200 to 900 nm. The spectra were compared with each other and one of at least 3 similar spectra is shown in this article.

X-ray Diffraction (XRD) Spectroscopy: XRD spectroscopy technique was used to identify the crystalline phase of AgNPs prepared by pomegranate peel extracts. The spectrum data were recorded at room temperature, using a BRUKER D8 ADVANCE X-ray diffractometer, (Bruker AXS, Karlsruhe, Germany), with monochromatic X-rays with a wavelength of 0.1542 nm generated by using Cu Kα source under emission current of 200 mA and a voltage of 40 kV. The diffraction patterns were collected in the range of 2θ = 5° to 90° with an incremental step size of 0.02° using flat plane geometry with 2 second acquisition time for each scan.

Hydrodynamic particle size distribution and surface charge: The hydrodynamic diameter and the zeta potential of the AgNPs in distilled water containing 10 mmol l^-1^ KCl were characterized by DLS using a Malvern Zetasizer Nano Measurements ZS-90 (Malvern Instruments, Worcestershire, UK).

Particle size of AgNPs in the dried state: Copper grid was submersed in AgNPs solution and allowed to dry at room temperature. The dried AgNPs were then characterized by a TEM instrument (HR-TEM, Jeol, JEM2100, Tokyo, Japan).

### 4.4. Bacterial Strains

Gram-positive *Staph. epidermidis* and *Staph. aureus* and Gram-negative *E. coli*, *Kl. pneumoniae*, *Pr. mirabilis*, and *Ps. aeruginosa* strains were obtained from the culture collection of the Department of Microbiology and Biotechnology, Faculty of Pharmacy, Delta University for Science and Technology.

### 4.5. Antibacterial Effect of Silver Nanoparticles by Agar Well Diffusion Method

The broth of bacteria was incubated at 37 °C overnight. The turbidity was adjusted to the approximately equivalent of 0.5 McFarland turbidity standards. The adjusted broth cultures were further diluted 1:200 in broth to obtain an inoculum density of bacteria ranging from 10^5^ and 10^6^ cell mL^−1^. Mueller–Hinton agar plate surfaces were inoculated by spreading bacteria over the entire agar surface by a swab. A hole of 6 to 8 mm diameter was punched with a sterile cork borer. A volume of 50 µL of the silver nanoparticles at a concentration of 50 mg mL^−1^ was introduced into each well. The plates were incubated at 37 °C overnight [46].

### 4.6. Coating of the Catheter with Silver Nanoparticles

One centimetre urinary catheter segments were dipped into a coating solution of a mixture of silver nanoparticles (50 mg mL^−1^) and mastic (50 mg mL^−1^) dissolved in 1 mL chloroform so that both internal and external surfaces of catheters were coated. Catheter pieces were dried overnight at room temperature on a sterile surface under the air of laminar flow. The pieces were washed using sterile distilled water to remove loosely attached coating materials from the surface of the coated segments. The pieces were then left to air dry under an aseptic condition in laminar flow [47].

### 4.7. Agar Diffusion Test for the Bacterial Inhibitory Effect of Coated Catheters

A total of 5 mL semisolid Mueller–Hinton agar was inoculated with 10^5^ and 10^6^ cells mL^−1^ and poured onto the surface plates containing previously poured 10 mL Mueller–Hinton agar. The pieces of catheters were dipped into the semisolid overlay and after setting the plates were left in the refrigerator for one hour to allow for the diffusion of silver ions into the agar medium. The plates were incubated at 37 °C and the inhibition zones surrounding the pieces of catheters were measured after 18 h [48].

### 4.8. Viable Count Evaluation of the Inhibitory Effect of AgNPs-Coated Catheters

Each piece of the catheter was put into a test tube containing 5 mL sterile nutrient broth containing about 10^5^ CFU mL^−1^ of a tested bacterium. The test tubes were incubated at 37 °C for 24 h. Pieces of the catheter were removed, washed three times in sterile distilled water, and placed into sterile 10 mL sterile saline. The tubes were sonicated in a sonicator bath (MCS Digital Ultrasonic Cleaner) for 5 min. Bacteria released in the saline were counted after serial dilution in sterile saline and spreading onto the dried surface of Mueller–Hinton agar plates. The plates were incubated for 24 h at 37 °C and then the colonies were counted. The tests were performed in triplicate and both positive and negative controls were included in the experiment [47].

### 4.9. Scanning Electron Microscope Analysis

Segments from AgNPs-coated and uncoated control catheters were exposed to bacteria in nutrient broth, as described above and washed by distilled water. The segments of the urinary catheter were fixed with 2% glutaraldehyde, followed by osmium tetroxide, tannic acid, and uranyl acetate. Ethanol dehydration was performed, and samples were sputter-coated with Au-Pd (60:40 ratio). The surface architecture of catheters was examined for biofilm formation using scanning electron microscope (XL3C SEM, Philips Research, Eindhoven, The Netherlands) [14].

### 4.10. Quantitate Determination of Silver Ions Released from Catheters

Pieces of silicon catheters coated with silver nanoparticles were impregnated in a degassed 50 mM phosphate buffer (pH 7.5) at 37 °C. The catheter was transferred every 24 h to a new tube containing a fresh solution of phosphate buffer. The amounts of the released silver ions were quantitatively determined by adding diluted hydrochloric acid and the turbidity produced was compared to a standard curve drawn by standard silver nitrate concentrations treated with diluted hydrochloric acid.

### 4.11. Statistical Methods

The experiments were carried out in triplicates. Variables were expressed as mean and standard deviation. IBM’s SPSS Statistics (Statistical Package for the Social Sciences) for Windows, version 25, 2017 was used for statistical analysis. The normality of data distribution was used to check the Shapiro–Wilk test. All tests were conducted with a 95% confidence interval and a *p*-value ≤ 0.05 was considered statistically significant. Kruskal–Wallis with Dunn’s post hoc analysis tests were applied for inter-group comparison of non-parametric continuous data.

## 5. Conclusions

In conclusion, the varnish of silicon catheters with AgNPs prepared by the extracts of the rind of the fruit of the pomegranate significantly inhibited bacterial colonisation of silicon catheters by six Gram-positive and Gram-negative bacteria. Therefore, catheters varnished with AgNPs could be a good candidate for the prevention of biofilm formation in vivo. We are currently investigating the use of catheters varnished with AgNPs for the prevention of biofilm formation in vivo.

## Data Availability

The data presented in this study are available on request from the corresponding author.
